# Simulated microgravity induces a cellular regression of the mature phenotype in *human* primary osteoblasts

**DOI:** 10.1038/s41420-018-0055-4

**Published:** 2018-05-10

**Authors:** Magda Gioia, Anna Michaletti, Manuel Scimeca, Mario Marini, Umberto Tarantino, Lello Zolla, Massimo Coletta

**Affiliations:** 1 Department of Clinical Sciences and Translational Medicine, University of Rome Tor Vergata, Rome, Italy; 2 Department of Ecological and Biological Sciences, University of Tuscia, Viterbo, Italy; 3 Department of Biomedicine and Prevention, University of Rome Tor Vergata, Rome, Italy; 4 Department of Systems Medicine, University of Rome Tor Vergata, Rome, Italy

## Abstract

Decreased mechanical loading on bones, such as prolonged bed rest and microgravity during space flights, leads to the development of an osteoporotic-like phenotype. Although osteoblast hypo-functionality is reported to be involved in the progression of bone pathological conditions, the cellular mechanisms of this process remain largely unknown. The combined application of mass spectrometry “–omics” and histochemical and ultrastructural approaches have been employed to investigate the effects of the gravitational unloading on *human* bone-cell biology. Here we show, ex vivo, that simulated microgravity (Sμg) on human primary osteoblasts (hpOB) induces an alteration of pro-osteogenic determinants (i.e., cell morphology and deposit of hydroxyapatite crystals), accompanied by a downregulation of adhesive proteins and bone differentiation markers (e.g., integrin beta-1, protein folding Crystallin Alpha B (CRYα-B), runt-related transcription factor 2 (RUNX-2), bone morphogenic protein-2 (BMP-2), and receptor activator of nuclear factor kappa-B ligand (RANK-L)), indicating an impairment of osteogenesis. Further, we observed for the first time that Sμg can trigger a transition toward a mesenchymal-like phenotype, in which a mature osteoblast displays an hampered vitamin A metabolism, loses adhesive molecules, gains mesenchymal components (e.g., pre-osteoblast state marker CD44), morphological protrusions (filopodium-like), enhances GTPase activities, which in turn allows it to acquire migrating properties. Although this phenotypic conversion is not complete and can be reversible, Sμg environment proves a plasticity potential hidden on Earth. Overall, our results suggest that Sμg can be a powerful physical cue for triggering ex vivo a dedifferentiation impulse on hpOBs, opening a new scenario of possible innovative therapeutical biomechanical strategies for the treatment of osteo-degenerative diseases.

## Introduction

Bone is a highly mechano-sensitive tissue, capable of undergoing rapid and robust rearrangement even in response to microscopic mechanical stimuli. Hence, cell mechano-transduction pathways are promising targets for new anabolic therapeutic strategies. So far, various biochemical factors are known to encourage osteoblast recruitment and osteogenesis^1–4^, whereas relatively little is known about how osteoblasts migrate/differentiate in response to mechanical signals.

Studies on stem cell differentiation have defined the principles of mechanobiology; cells sense extracellular stiffness through contraction of the actomyosin cytoskeleton, regulating the suitable response through focal adhesion, rho-GTPase signaling, cytoskeletal contractility, and nuclear rearrangement processes^5,6^. Empirical evidences report that mechanical unloading impairs osteoblasts differentiation of bone marrow mesenchymal stem cells, thus inhibiting osteogenesis^7,8^.

Differentiation process is tightly intertwined to cellular motility, since they both involve (i) a peculiar actin organization forming specific cell-protrusions (such as lamellipodia, filopodia, or blebs)^9,10^, (ii) a specific proteolytic set of enzymes^11–13^, and (iii) adhesion proteins (e.g., cluster of differentiation proteins and integrins)^11–16^.

Although humans have limited regenerative capacity, in principle, a mechanical induction of dedifferentiation may be a logical strategy to promote regeneration in tissues that lack osteogenic ability^17–19^.

Unfortunately, neither mechano nor biochemical mechanisms of osteoblast dedifferentiation are comprehensively known. Apparently, cellular plasticity (i.e., cellular susceptibility to reprogramming) decreased during evolution processes. By contrast to what happens in fish biology^20^, in mammals mature osteoblasts do not contribute to bone repair^21^. However, some evidences are documented (in adult skull-cap-derived cells and pediatric osteosarcoma)^22,23^, suggesting that a dedifferentiation potential can still be conserved in mammalian mature osteoblasts.

Retinoic acid (RA), a metabolite of vitamin A, plays a central role in cellular dedifferentiation^24^, and it may also play a role in mechano-biology. Retinoids are considered promising leading compounds in differentiation therapy strategies^25^ as they are reported to inhibit osteoblast dedifferentiation at cellular and molecular levels^26,27^.

Although on Earth osteoblasts have evolved and constantly behave in the presence of gravity, experiments performed in microgravity (either off planet or simulated) have shown how the gravitational force is a biological stressor of bone physiopathology^14,18,28–31^. In particular, investigations on human osteoblastic cell lines report that microgravity inhibits the osteogenesis across all differentiating morphological and molecular features (e.g., cell cytoskeletal organization and adhesion, bone phenotypic markers, alkaline phosphatase (ALP), hydroxyapatite (HA) crystals, matrix metalloproteinases)^14,32–37^.

The present study has been carried out on human primary osteoblasts (hpOBs) from healthy donors with the aim of revealing effects of Sμg on cellular response. The investigation has been undertaken by ultra-structural, immune-cytochemistry, cell biochemistry, and quantitative mass spectrometry (MS) proteomic and metabolomic approaches to assess whether the Sμg-induced loosening of obsteoblast mature phenotype were correlated with hypo-functional cellular aspects.

Overall, present data indicate that upon Sμg treatment hpOBs do not just lose the mature morphological phenotype but they also become biochemically hypo-functional cells. Further, we report, for the first time, that under Sμg hpOBs display a reversal of mature-osteoblast differentiation features, which renders cells capable of healing a wound in vitro.

## Results

### Simulated microgravity alters the morphological phenotype of mature hpOBs

We employed random positioning machine (RPM) machine for reliably mimicking experimental cell culture conditions actually occurring in space^38^. HpOBs from healthy donors were selected for monitoring the specific response to the weightless treatment.

**Fig. 1:**
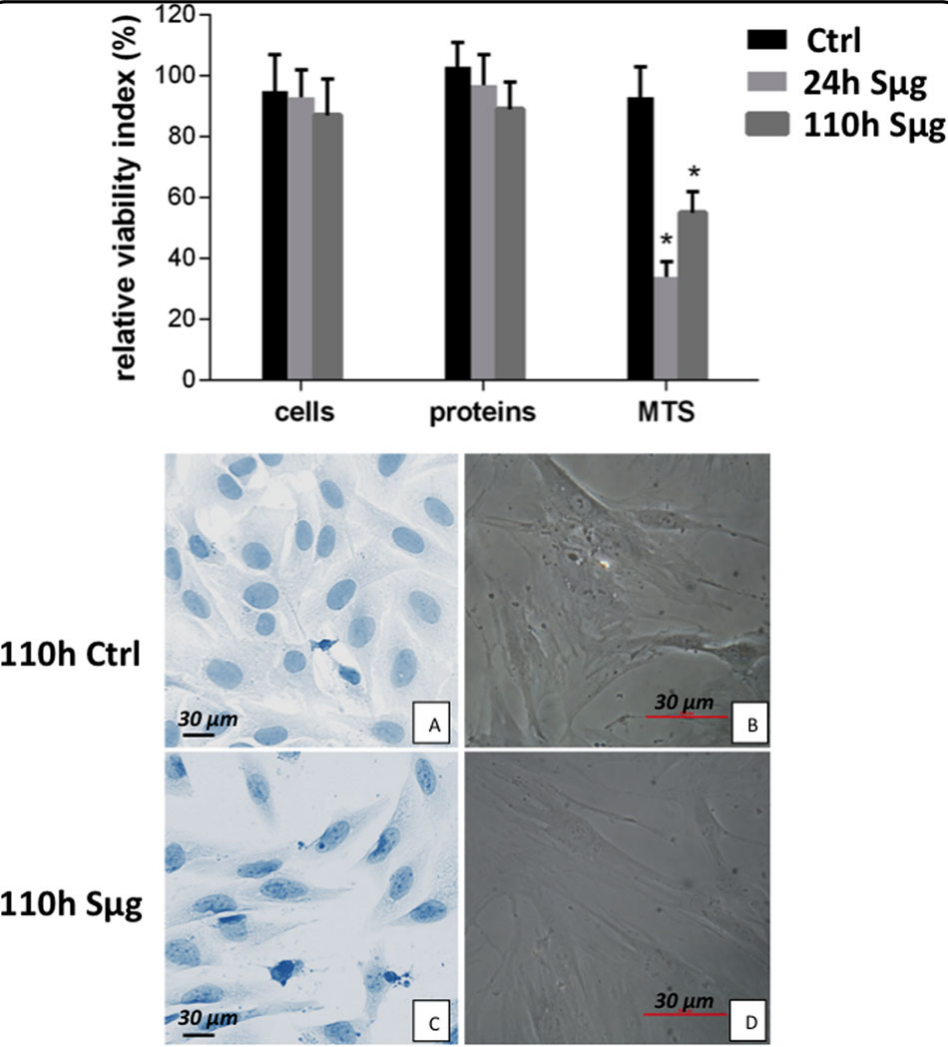
Effect of simulated microgravity on hpOP cells. Upper panel: The relative effect of microgravity treatment on viability was expressed as relative cell viability index. The results were expressed as percentage of (i) number of cells with intact plasmamembrane (trypan blue impermeable cells), (ii) cell protein content (BCA absorbance), and (iii) metabolic active cells (MTS absorbance). Histograms represent mean ± SD (*n* = 6), black columns refer to control samples, light gray columns represent 24 h, and dark gray represent 110 h-Sμg-exposed cells. A one-way analysis of variance (ANOVA) was performed and followed by Tukey’s honestly significant difference test (*n* = 6 for each experimental conditions). Significant decrease from respective control values at *p* < 0.05 is denoted as *. Lower panel: Morphology of differentiated hpOB cells. **a**, **b** Toluidine blue staining pictures from inverted microscope (Leika) of cells under terrestrial gravity display a typical aspect of mature osteoblasts, respectively. **c**, **d** Pictures from up-right microscope (Nikon) of hpOBs exposed to 110 h Sμg. Image shows that Sμg hpOBs do not retain the mature phenotype of osteoblasts acquiring a spindle-shaped cell morphology

In our experimental set-up of simulated microgravity, osteoblasts retain cell viability, as variations in cell number (i.e., percentage of trypan-blue impermeable cells) and protein content (i.e., percentage of BCA absorbance) are not appreciable (Fig. 1), whereas a dysfunction of the mithocondrion metabolism (reported by formazan 3-(4,5-dimethylthiazol-2-yl)-5-(3-carboxymethoxyphenyl)-2-(4-sulfophenyl)-2H-tetrazolium (MTS) formation) is detected, as also previously reported by metabolomic and proteomic approaches^39^. Similarly to what reported for rat cells^32^, our results show that also for hpOBs the in vitro differentiation is impaired by Sμg. Here we document a gravitational-sensitive response for human purified osteoblasts after a 110 h-treatment of Sμg. Consistently, cell inspection by inverted and up-right microscope revealed a morphological shift from flat-shape to spindle-shape (Fig. 1), clearly showing that the majority of cellpopulation treated with Sμg displays a spindle-shaped cell morphology (Fig. 1b, d) rather than the cuboidal cell shape typical of the mature osteoblast phenotype (Fig. 1a, c). A similar morphological cell shift occurs also after a shorter RPM treatment (i.e., 18 h).

**Fig. 2:**
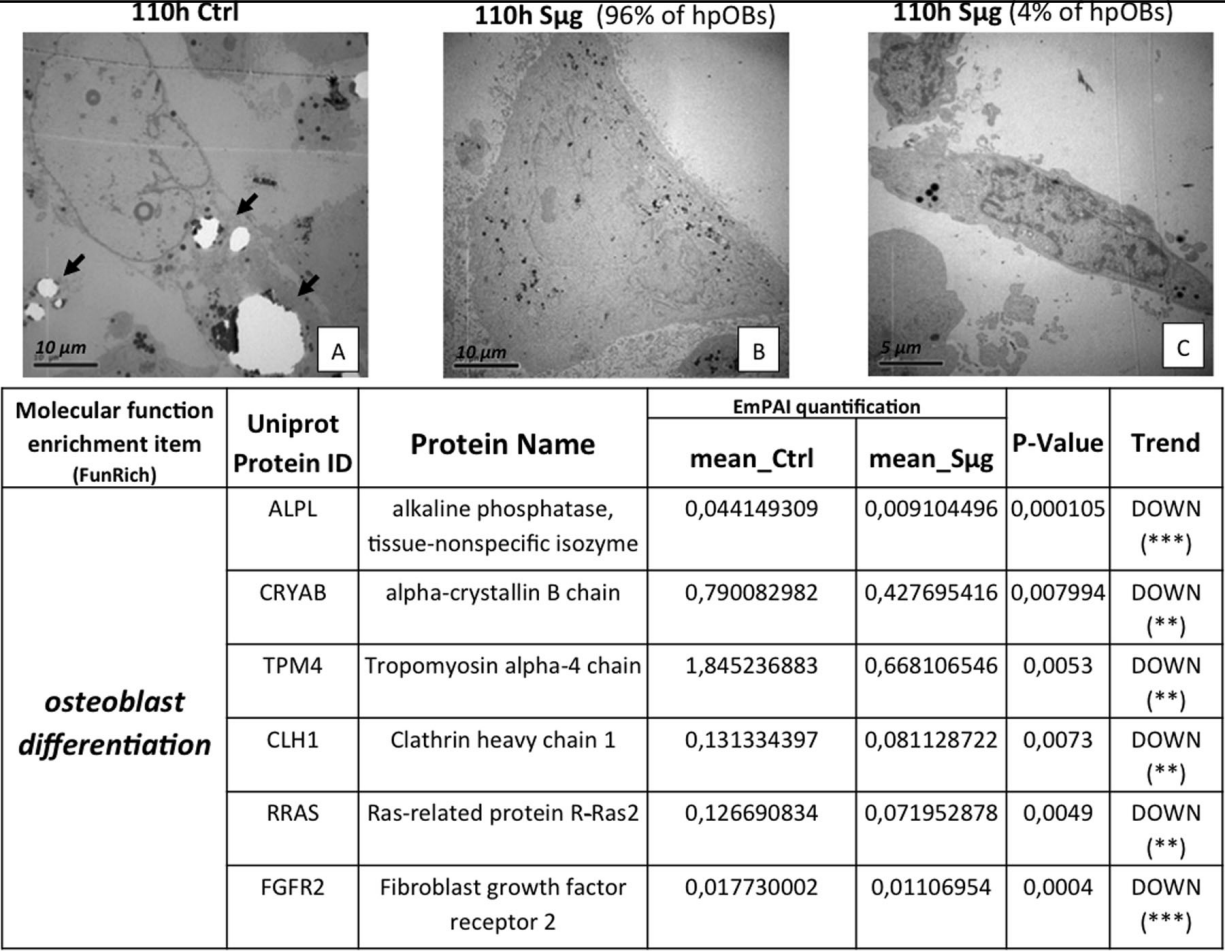
Loosening of osteoblast phenotype induced by Sμg. Upper panel: TEM micrographs of hpOBs. **a** Normo-gravity control mature hpOBs micrograph taken at 110 h time point. Ultrastructural image show osteoblast cell with intracellular granules of HA crystals (highlighted by arrows). **b** Mature osteoblasts exposed to 110 h Sμg. Image represent 90% of cells, which show large cells without both profuse endoplasmic reticulum and intracellular granules of HA crystals. **c** Representative TEM micrograph of 4% of cell after 110 h Sμg. Images display a “mesenchymal-like” cell induced from 110 h-Sμg treatment. Lower panel: Downregulation of pro-osteogenic proteins. The tables reports differential spectral-counts between normo-gravity and Sμg conditions. Protein content was estimated as normalized EmPAI values as percentage according to Shinoda et al. The table shows proteins differentially regulated in abundance with statistical significance. *p < 0.05; **p < 0.01; ***p < 0.001, and unique proteins in normo-gravity (UN) and microgravity (UM)

Moreover, as expected, a decreased amount of HA crystals, which are laid down during the osteogenesis, is shown by the comparison of transmission electron microscopy (TEM) images between hpOBs cultured under Sμg and normo-gravity conditions (Fig. 2a, b, respectively). Therefore, under Sμg cells produce less bone deposit material than control cells, confirming that the Sμg reduces the osteoblast differentiation rate^33–35^.

### Simulated microgravity alters the “biochemical phenotype” of mature hpOBs

We investigated by analytic MS-proteomic alterations of cell functionality, comparing the proteome for molecular reporters of hpOB differentiation (i.e., biochemical phenotype) between cells exposed to Sμg and those left under normo-gravity for 110 h.

Exponentially modified protein abundance index (emPAI, a label-free relatively quantitative method) revealed that the decreased hpOB differentiation was accompanied by a reduction of some of key proteins involved in the skeletal mineralization process (*p* < 0.01) (Table in Fig. 2). Supplementary Table 1 resumes the trends of protein levels which were altered by Sμg, specifying the biological function of every single protein (according to the Gene ontology protein classification). Of particular note is the decrease of the osteogenic markers, such as alkaline phosphatase tissue-nonspecific isozyme (ALPL), and the tissue-specific proteins related to protein folding Crystallin Alpha B (CRYαB)^40^ (Table in Fig. 2, for further information see next sections), indicating that upon Sμg hpOBs do not just lose the mature morphological phenotype but they also become biochemically hypo-functional cells.

#### Simulated microgravity-induced dedifferentiation

A deeper ultrastructural morphological inspection by TEM on cells exposed to Sμg confirmed an overall reversion of the differentiation determinants; in particular, a small but significant percentage of cells (about 4%) reverts back to a less-differentiated stage, displaying a mesenchymal cell-like phenotype (Fig. 2c). No similar cell phenotype change was detected in cells cultured under normo-gravity conditions. These evidences suggest that the Sμg-induced cell hypo-functionality could stem from a dedifferentiation process rather than just a simple slowing down of the osteogenic process.

**Fig. 3:**
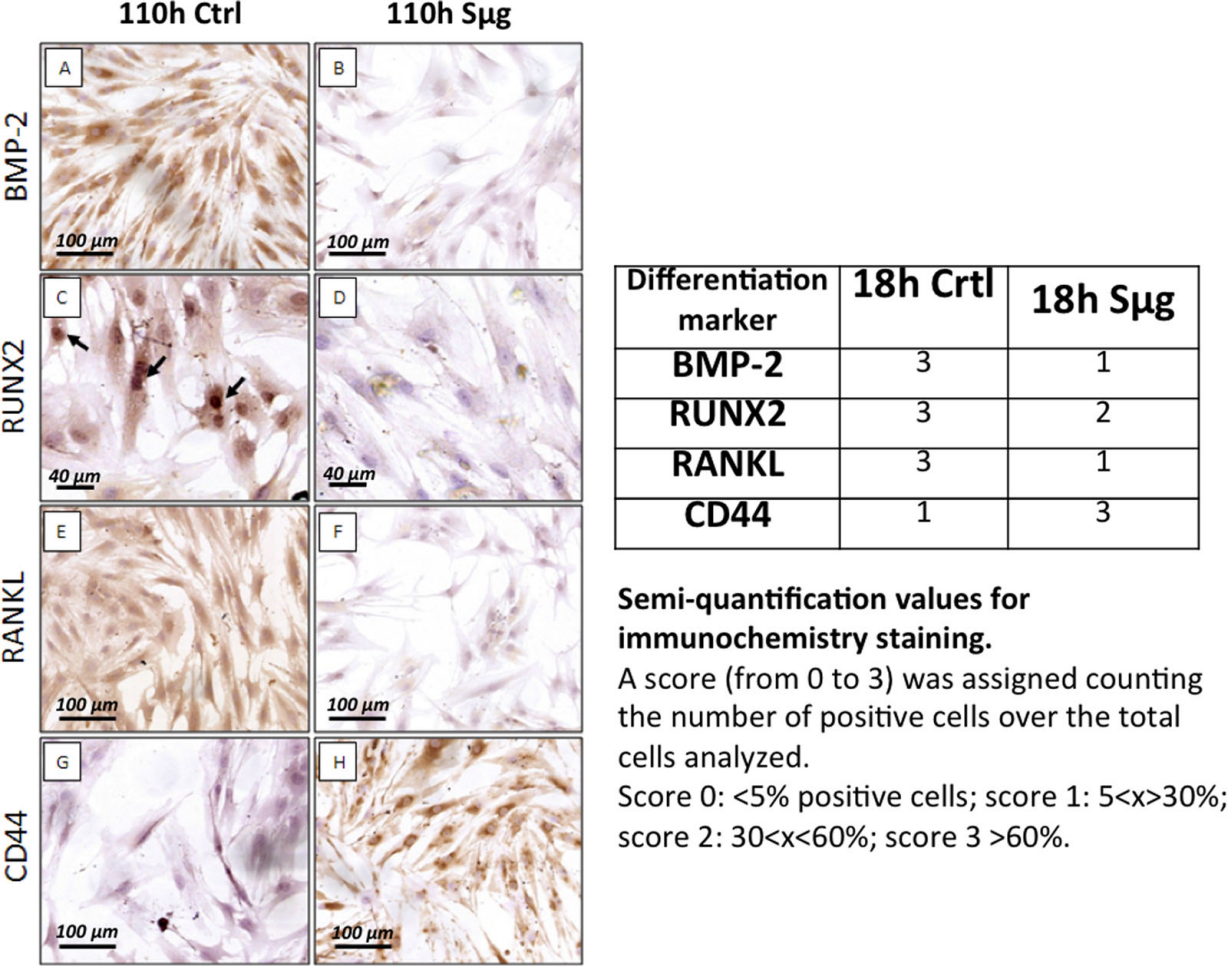
Sμg reduces hpOB differentiative potential. Left panel: Immuno-cyto-chemical staining of differentiation markers on hpOB cells. **a** Image shows numerous well-differentiated BMP-2-positive osteoblasts in normo-gravity condition. **b** After 24 h Sμg treatment, osteoblasts lose the expression of BMP-2. **c** Nuclear RUNX2 expression (arrows) in osteoblasts under terrestrial gravity. **d** After 24 h Sμg expostition, a decreases of RUNX2 nuclear expression is observed. **e** In normo-gravity condition more than 95% of osteoblast express RANKL. **f** After 24 h Sμg expostition, osteoblasts downregulate the expression of RANKL. **g** Image shows rare CD44-positive osteoblasts in normo-gravity. **h** 24 h Sμg treatment induces the expression of CD44 in osteoblasts. Right panel: Semi-quantitative analysis of the variation of osteoblast differentiation markers on hpOBs. The immuno-histochemical reaction was evaluated by assigning a score from 1 to 3 according to the number of positive cells. Semi-quantification values refer to the 18 h incubation time. Dashed lines highlight the place where the scratch occurred. Dotted lines indicate wound leading edges. Phase contrast images (microscopic magnification 4×)

An immune-cytochemistry approach was employed to characterize the dedifferentiation process looking at the osteogenic differentiative markers, such as the bone morphogenic protein-2 (BMP-2), runt-related transcription factor 2 (RUNX-2), receptor activator of nuclear factor kappa-B ligand (RANK-L), and the pre-osteoblast state marker, named cluster of differentiation protein 44 (CD44)^41^. Figure 3 indeed seems to support the occurrence of the dedifferentiation process, showing that the Sμg treatment induced: (i) the downregulation of BMP-2, RUNX-2, and RANK-L, and (ii) the upregulation of CD44 (Fig. 3). Of note, a similar immune-cytochemistry trend was apparently observed even upon an incubation time shorter than 110 h. Thus, a semi-quantitative imaging analysis (according to HIC scoring), performed on specimens incubated for 18 h, proved that the differentiation markers (i.e., BMP-2, RUNX-2, and RANKL) decreased also upon a much shorter exposure (i.e., 18 h) (Table in Fig. 3).

### Cell migration under simulated microgravity induces a regenerative response in hpOBs

Assuming that changes in cell locomotion were an indicator of a transition phase across the processes of osteogenic differentiation, we believed relevant to investigate how cell migration was affected by Sμg. Cell motility was examined by 2D wound healing assay which is an established method for assessing cancer aggression for osteosarcoma cells. Therefore, first we performed experiments on SAOS II osteosarcoma cells (osteoblast-model cell line commonly used as reference) (see Supplementary sections), and then similar procedures were applied to hpOBd.

**Fig. 4:**
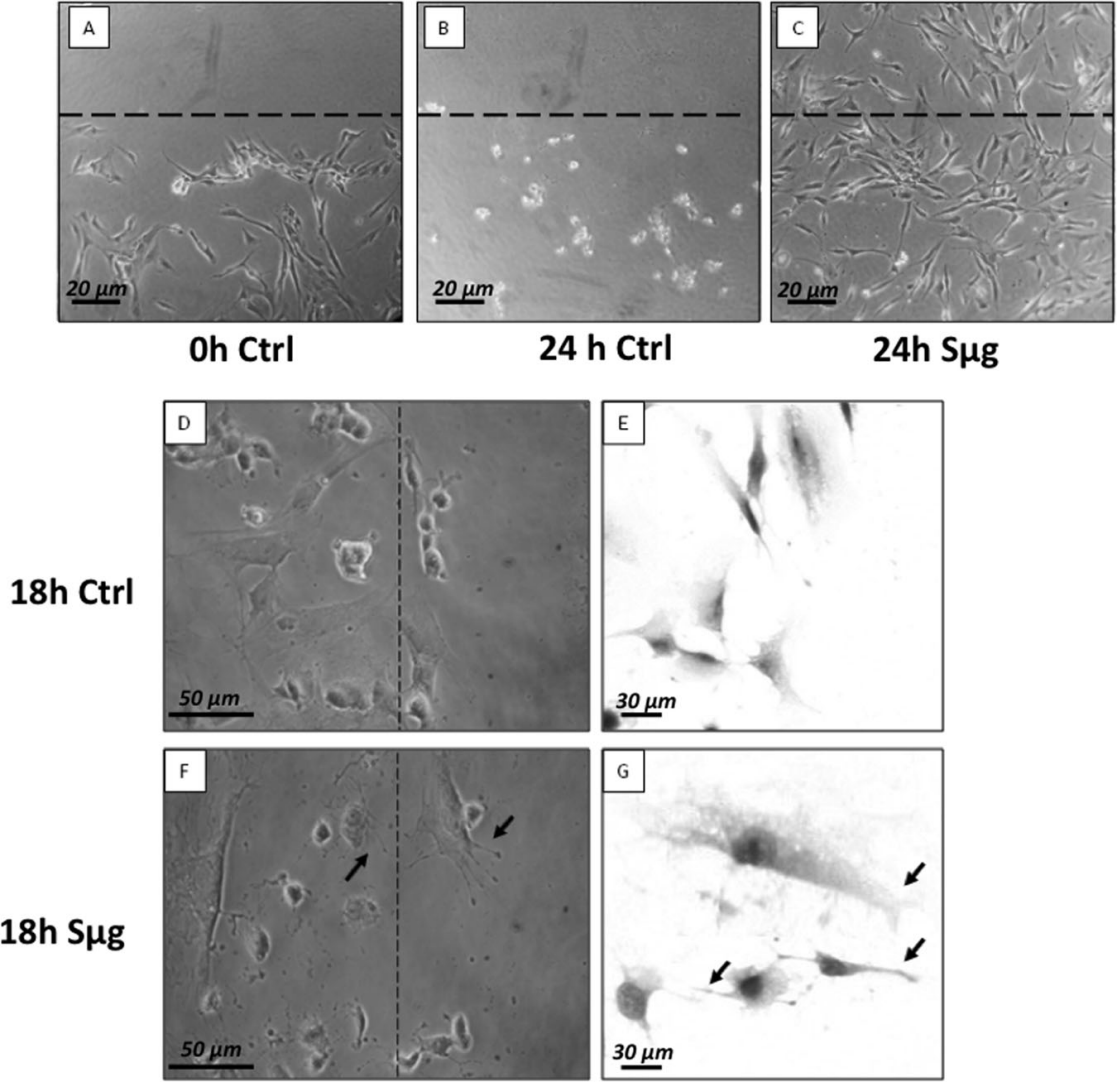
Effect of sμg on hpOb motility. Upper panel: Wound assay on hpOB cells normo-gravity vs. Sμg. **a** Scratched cell monolayer recorded at t0. **b** The wound after 24 h under normo-gravity. **c** The wound after 24 h Sμg exposition. Dashed lines highlight the place where the scratch occurred (microscopic magnification 4×). Lower panel: Cell migrating front: ZOOM of hpOBs at 18 h after the wound: normo-gravity vs. Sμg. **d**, **e** Micrograph of hpOBs close to leading edge under normo-gravity conditions from upright and inverted microscope, respectively. Images display no/rare cellular structures compatible with cell protrusions (filopodia or lamellipodia). **f**, **g** Images show osteoblasts next to leading edge after 18 h microgravity incubation from upright and inverted microscope, respectively. Arrows indicate the presence of several lamellipodia or filopodia-like protrusions. Toluidine blue staining in pictures from inverted microscope

The osteotropism effect of Sμg on hpOBs was examined by 24 h monitoring: thus, right after the wound was procured, the scratched hpOB monolayers were (or not) treated with Sμg. Figure 4a shows fully differentiated hpOBs layer at the time of the wound (t0), Figure 4b displays that after 24 h under normo-gravity the wound is not visible anymore as for cells detached and died. Thus, after the wound scratch, cell migration did not occur for the stationary normo-gravity cultured and almost all cells detached and died within the 24 h (Fig. 4b).

On the other hand, when the scratched hpOB monolayer was exposed to Sμg for 24 h, an astonishing regenerative impulse was observed. Figure 4c shows a complete coverage of the wounded area by loosely connected population of cells, suggesting that the Sμg treatment unveiled the hidden cellular plasticity (i.e., a cellular susceptibility to reprogramming) of hpOBs, which in Sμg conditions mimic migration of fibroblasts on Earth.

In view of the reported anabolic effect of osteoblasts secretome as co-ordinator of bone formation, particularly in response to mechanical stimulation^3^, we tested whether the Sμg-conditioned medium could recover the wound hpOBs monolayer left under normo-gravity conditions. However, cell-imaging analysis did not appreciate any meaningful improvement of the repair process under normo-gravity conditions in the presence of Sμg hpOB conditioned media (data not shown). It demonstrates that the anabolic Sμg response cannot be simply reproduced by a paracrine signal.

A closer look at the wounded leading edges (Fig. 4) indicates that (i) transition of cells induced the losening of the osteoblast phenotype and the acquisition of fibroblast-like features, (ii) cells display actin-based protrusions such as pseudopodia (similar to lamellipodia and filopodia but not to blebs) (Fig. 4f, g), which mediate the migration of mesenchymal cells^10^ (see also supplementary Fig. 1).

### Simulated microgravity effect on the “motility proteome”

As reported above proteins concurrently involved in migration and differentiation were found downregulated in Sμg proteome (see Fig. 2). To further investigate the gravitational impact on cell motility proteins the MS-proteomic study was addressed to three classes of proteins, namely (i) adhesion proteins, (ii) protrusion dynamic networks, and (iii) rhoGTP signaling cascade, which are collectively reported to play important roles in migration and in biophysically induced cell response^5,42^. Within the “motility proteome” only proteins which displayed protein levels (emPAI) altered by Sμg were examined and classified according to Fun Rich (Platform 3.0). Spectral counts are reported in Table 1, whereas Supplementary Table 1 resumes trends of protein levels after the Sμg treatment, specifying the biological functions known for each protein.

**Table 1:** “Motility” proteins displaying differential spectral-counts between normo-gravity and microgravity conditions

Molecular function enrichment item (FunRich)		Uniprot protein ID	Protein name	EmPAI quantification		*P*-value	Trend
				mean_ctrl	mean_Sμg		
(i) Adhesion proteins		TLN 1	Talin	0,188123068	0.070200944	2,11E-06	DOWN (***)
		ITGB 1	Integrin beta 1	0,06516658	0,044034984	0,0507549	DOWN
		ITGA3	Integrin alpha-3	0,006907204	0,004317346	0,0155	DOWN (*)
		ZYX	Zyxin	0,131873677	0,068814834	0,039799	DOWN (*)
(ii) Protrusion dynamic actin network	Arp2/3 complex	ARPC 5	Actin-related protein 2/3 complex subunit 5 (p16)	0,074171194	0,162733776	0,046479	UP (*)
		ARPC 3	Actin-related protein 2/3 complex subunit 3 (p21)	–	0120347326	–	UM
		ARPC 2	Actin-related protein 2/3 complex subunit 2 (p34)	0,167532621	0,065708433	0,000261	DOWN (***)
		ACTR 3	Actin-related protein 3 (ARP3)	0,049531658	0,033574567	0,012523	DOWN (*)
		ACTR 2	Actin-related protein 2 (ARP2)	0,051803205	–	–	UN
		ARPC 4	Actin-related protein 2/3 complex subunit 4 (p20)	0,040176784	0,172100443	6,68E-08	UP (***)
		ARPC 1B	Actin-related protein 2/3 complex subunit 1B (p41 ARC)	0,022004409	–	–	UN
	Cytoskeletal organization proteins	ENAH	Protein enabled homolog	0,011537141	0,007866219	0,022371	DOWN (*)
		VASP	Vasodilator-stimulated phosphoprotein	–	0,014752477	–	UM
		CFL 1	Cofilin-1	0,742005965	0,304803386	0,024149	DOWN (*)
		PFN 1	Profilin	2,096222416	1,400267715	0,044221	DOWN (*)
		FSCN 1	Fascin	–	0,027958567	–	UM
		RDX	Radixin		0,074884277	–	UM
		MYH 10	Myosin-10	0,052986425	–	–	UN
(iii) RhoGTP signaling		RHO A	Transforming protein rhoA precursor	–	0,109655994	–	UM
		RHO G	Rho-related GTP-binding protein RhoG precursor	–	0,019540636	–	UM
		RAC 1	Ras-related C3 boyulinum toxin substrate 1	–	0,04918568	–	UM
		ARHGDI A	Rho GDP-dissociation inhibitor 1	0,063887075	0,044089322	0,04036	DOWN (*)
		ARHGDI B	Rho GDP-dissociation inhibitor 2	0,035981062	–	–	UN
		ARHGEF 18	Rho guanine nucleotide exchange factor 18	0,0087225	–	–	UN
		RHG01	Rho GTPase-activating protein 1	–	0,00929341	–	UM
		ROCK 2	Rho associate protein kinase 2	0,010915361	–	–	UN

Protein content was estimated as normalized emPAI values express as percentage according to Shinoda et al. The table shows proteins differentially regulated in abundance with statistical significance

*p < 0.05; **p < 0.01; ***p < 0.001, and unique proteins in normo-gravity (UN) and microgravity (UM)

(i) Among the adhesion proteins we detected downregulation of two integrin subunits (i.e., integrin beta-1 (ITGB1 or CD29) and integrin alpha-3 (ITGA3 or CD49)), as well as of two integrin-binding proteins (i.e., tailin and zyxin) suggesting, as previously reported^14^, that a suboptimal integrin-ligand level might reflect an inefficient cell adhesion (Table 1 and Supplementary Table 1).

(ii) Concerning the proteins involved in the formation of dynamic cell protrusions, the MS quantification revealed a Sμg dysregulation of proteins involved in the cytoskeletal dynamic formation of cell protrusions (i.e., lamellipodia and filopodia). In particular, these include actin-interacting regulatory proteins, such as the multifunctional organizer Arp2/3 complex, a conserved multi-protein complex that facilitates the formation of actin-based protrusions essential for cell motility. It must be outlined that the Arp2/3 complex is formed by seven members, three of which, namely, ARP2, ARP3, and p34/ARPC2, were reduced by Sμg, whereas other three, namely, p21/ARPC3, p20/ARPC4, and p16/ARPC5, displayed an upregulation. Similarly, a dysregulation was observed for another set of cytoskeletal organization proteins, such as Enah, cofilin, profilin1, fascin, radixin, myh10 (all of which were downregulated); conversely, vasodilator-stimulated phosphoprotein (Vasp) was upregulated (Table 1 and Supplementary Table 1). Overall, these data indicate that the dynamic formation of distinct actin-dependent structures is sensible to the gravitational force, making it reasonable to speculate that under Sμg conditions the peculiar assembly/disassembly coordination could influence the kinetics of cell migration.

(iii) With regard to rhoGTP signaling cascade, Sμg favors Rho GTPase activities, as these enzymes (namely, RHOA, RHO G, and RAC1) were uniquely found in the Sμg proteome, whereas a concurrent downregulation (or the complete absence) of their negative regulators (namely, Rho GDP Dissociation Inhibitor Alpha (ARHGDIA), Rho GDP Dissociation Inhibitor Beta (ARHGDIB), and Rho/RAC GTP exchange factor (ARHGEF18)) were detected (Table 1). However, ROCK, one of the downstream target of rhoA, was downregulated, suggesting that ROCK may not be essential for migration under Sμg conditions (Table 1 and Supplementary Table 1).

### Effect of simulated microgravity on proteins and metabolites of the vitamin A metabolism

**Fig. 5:**
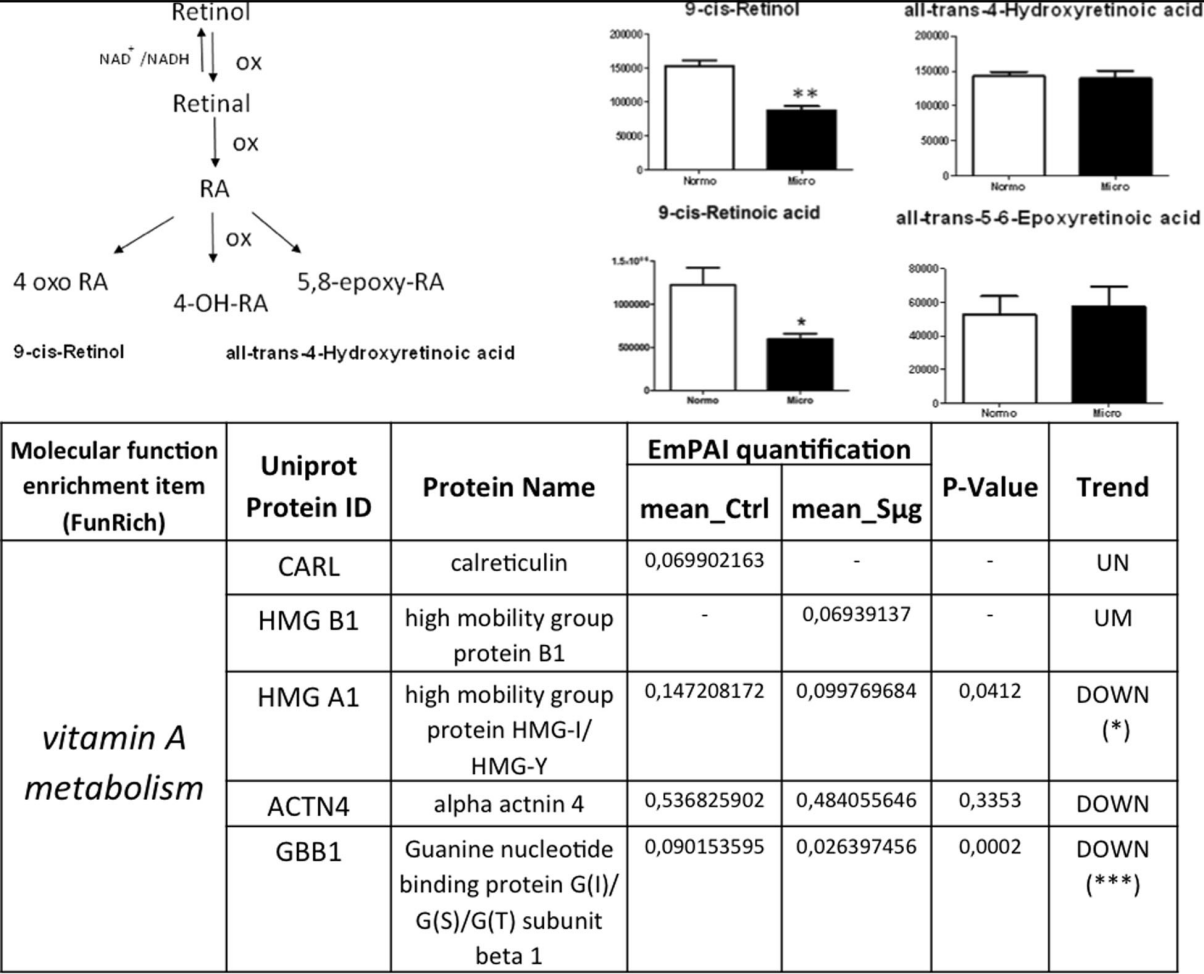
Simulateded microgravity treatment hampers vitamin A metabolism on hpOB cells. Left upper panel: A sketch of metabolism of vitamin A (retinol): Physiological RA, the most bioactive form of the vitamin A, is biosynthesized from dietary retinol (vitamin A) through two oxidation steps and it is catabolized by additional oxidizing steps that generate several inactive species (i.e., 4-OH-RA, 4-oxo-RA, and 5,8 epoxy RA). Right upper panel: Variation in the levels of metabolic intermediates in Vitamin A metabolism. Values are mean ± SD (*n* = 9) of normo-gravity (white columns) and microgravity (black columns) metabolites. *p < 0.05; **p < 0.01; ***p < 0.001. Lower panel: Spectral count-relative abundance for “vitamin A proteome” on hpOBs between under normo-gravity and Sμg conditions. Protein content was estimated as normalized emPAI values as percentage according to Shinoda et al. The table shows proteins differentially regulated in abundance with statistical significance. *p < 0.05; **p < 0.01; ***p < 0.001, and unique proteins in normo-gravity (UN) and microgravity (UM)

Since RA is known to be one of the major small molecular regulators of osteoblast dedifferentiation^27^, we have examined the vitamin A pathway. In order to detect any Sμg-induced perturbation on vitamin A metabolism, a quantitative metabolomic comparison was carried out between active and inactive metabolites of vit A (whose steps are sketched in Figure 5 left upper panel). The comparative analysis showed that after an exposure of 110 h to Sμg both precursors and the active forms of vitamin A (i.e., retinol and RA) were decreased (see Fig. 5a, b), without affecting the cellular levels of vitamin A inactive forms (Fig. 5c, d). Searching for proteins directly related to the vitamin A pathway within the hpOB proteome, none of the peak lists could be assigned to direct enzymes nor to receptors involved in vitamin A metabolism, likely because these proteins are opaque to the employed experimental MS approach. However, proteins indirectly related to this metabolism (according to Funrich annotation) were identified by Mascot search. Interestingly, among these proteins, only those negatively affecting the vitamin A metabolism were upregulated (namely, calreticulin (CALR), which negatively regulates RAR function, and High Mobility Group Box 1 (HMGB1), whose release is inhibited by RA). Consistently, High Mobility Group Box 1 (HMGA1) and GBB1 guanidine were downregulated, as expected for a dampened vitamin A metabolism (see Fig. 5 lower panel).

## Discussion

The absence of gravity is a biological stressor whose impact on biological systems is far from being defined; its role in determining cellular functions can be cheaply investigated on earth with Sμg. Bone is a highly mechano-sensitive tissue, so that even a healthy bone can rapidly develop an osteoporotic-like phenotype upon prolonged exposure to unloading conditions, as it is experienced by astronauts during space-flights or by patients forced to bed rest. Although a hypo-function of the osteoblasts is reported to be involved in the progression of unloading-induced osteoporosis, the gravitational stress response of *human* not-genetically-transformed cells remains largely unknown.

Previously on hpOBs, we reported that Sμg changes cell metabolism, as revealed by an increased glycolysis pathways and by a severe impairment of the Krebs cycle pathway associated to a decrease of the malate-aspartate shuttle^39^. This was related to a significant alteration of chain electron transport of respiratory proteins, mainly affecting complex III^39^. Thereby suggesting that Sμg suppresses bone cell functions through a prominent dysregulation of mitochondria, which impairs the energy state and antioxidant capacity of hpOB cells.

Here we examined the effect of weightless conditions on osteogenesis, comparing cellular morphology, proteome profiling, metabolism, and cell motility between hpOBs exposed to Sμg vs. those kept under normo-gravity conditions. Our data show that upon Sμg treatment hpOBs (i) assume a spindle-shape morphology, characteristic of a less differentiated phenotype, (ii) decrease the production of HA crystals (laid down during the ossification process) (Fig. 2a, b), and (iii) reduce osteogenic differentiation markers (e.g., ALPL, CRYα-B, Runx-2, Rank-L, and BMP-2) (Fig. 3). A similar behavior was also reported by others through different experimental approaches^32–36^, confirming that Sμg significantly impairs osteoblastic differentiation.

Our findings show that the Sμg affects the hpOBs not just slowing down their differentiation process, but also inducing a phenotypic regression (accompanied to a loosening of pro-osteogenic specialized functions). Here we report for the first time that under Sμg conditions hpOBs display a transition process toward a mesenchymal-like phenotype, in which a mature osteoblast cell loses levels of adhesive proteins (see Table 1 and Supplementary Table 1), enhancing mesenchymal-like components (Figs. 2c, 3g, h), which allows it to acquire motility properties. Although this phenotype conversion can be reversible and often incomplete^43,44^, its occurrence is certainly a demonstration of a cellular regenerative potential. This observation is of particular interest, since under normo-gravity conditions mature-osteoblasts, though conserving a dedifferentiation potential^22,23^, do not participate to bone-healing neither in vitro nor in bone fracture repair processes in vivo (Fig. 4)^21,45^. Conversely, under Sμg conditions, hpOB cells show an unusual degree of plasticity as all of them revert to a prior developmental stage, and 4% of them switch to a mesenchymal-like phenotype.

Therefore, we can reasonably assess that hpOB receive an impulse to dedifferentiate by gravitational unloading, probably as a consequence of a metabolic/mitochondrial perturbation (Fig. 5)^39^. The Sμg dedifferentiation impulse is also consistent with the overall metabolic profile observed in hpOB exposed to Sμg^39^. Similarly to what reported for the metabolism of dedifferentiated cells^46,47^, we previously reported for osteoblasts under Sμg an enhanced glycolysis associated to an increased production of lipids and nucleotides, which are followed by a reduction of the Krebs cycle^39^. Interestingly, in Sμg conditions the reduced differentiation phenotype was not accompanied by a proliferative state^39^, supporting that the metabolic changes are compatible with a dedifferentiation switch. Nevertheless, a closely related developmental regression to a less differentiated cell phenotype was also reported for tumors and may contribute to metastasis^9,43,44^, indicating that the Sμg-induced dedifferentiation process deserves further studies studies to understand more accurately the mechanism underlying these complex changes for developing effectively novel biomechanical strategies in medicine.

In addition, the analysis of metabolites and protein profiling on the metabolism of vitamin A (i.e., retinol and RA), one of the major small molecular suppressors of osteoblast dedifferentiation^20,26^, revealed that RA metabolism is dampened under Sμg conditions (see Fig. 5). Thereby, in view of the inhibitory effect of RA on the osteoblast dedifferentiation^26,27^, a realistic hypothesis can be that the Sμg-induced dedifferentiation is triggered by low levels of bioactive vitamin A, even though we cannot rule out the possibility of additional factors (e.g., NFkB and BMPs).

Additional results which support that the dedifferentiation can be triggered by a Sμg exposure come from the alteration of cell migrating ability induced by Sμg treatment. During osteoblast differentiation, cells pass through several stages where cell morphology and biochemical phenotype change remarkably, overall influencing cell migration abilities. As a result, at the end of the differentiation process osteoblasts stop to migrate turning into the mature phenotype^2^. However, the exposure to Sμg induces a reversion of mature cell phenotype with upregulation of CD44, allowing hpOBs to acquire a resistance to death and motility properties that make wound healing possible in vitro (Fig. 4).

Since it is recognized that the gravitational force influences movement kinematics, it is not surprising that under Sμg cell motility was affected by a decrease of adhesive proteins and an increase of the numbers of cell protrusions with respect to normo-gravity control counterpart. In particular, the Sμg suboptimal integrin level of ITGB1 and ITGA3 (Table 1) might reflect an inefficient cell adhesion, as also reported by others^14^. Macroscopically, migration under Sμg conditions takes place through isolated rather than clustered cells, and it occurs through morphological protrusions which explore their surroundings by polymerization of the structural protein actin into filaments (i.e., protrusions dynamic networks), being morphologically compatible with filopodia and/or lamellipodia, but not with blebs (Fig. 4). Moreover, according to the Schafer’s model for the growing ends of actin filaments^48^, the unique presence of the promoters of actin filament elongations (i.e., Vasp, fascin, and radixin; Table 1 and Supplementary Table 1) in the Sμg proteome suggests that in these conditions filaments are filopodium-like. Thus, our findings indicate that Sμg triggers an osteotropic effect on human primary cells hpOBs, which induces a “survival impulse” avoiding cells from detaching (Fig. 4). This was also supported by the increased number of morphological protrusions (most likely filopodia) (Fig. 4) and by the dysregulation of levels of proteins related to protrusion dynamic networks and upregulation of GTPases activities (Table 1), which collectively play important roles in the biophysically induced cell response and migration^5,42,49^.

Notably, although the hpOBs secretome on Earth, particularly in response to mechanical stimulation, is reported to provide anabolic effects^3^, the conditioned medium from hpOBs exposed to Sμg could not rescue the repair process after wound scratch in hpOBs kept under normo-gravity conditions, thus indicating that the benefic effect of Sμg cannot be simply reproduced by a paracrine signal.

**Fig. 6:**
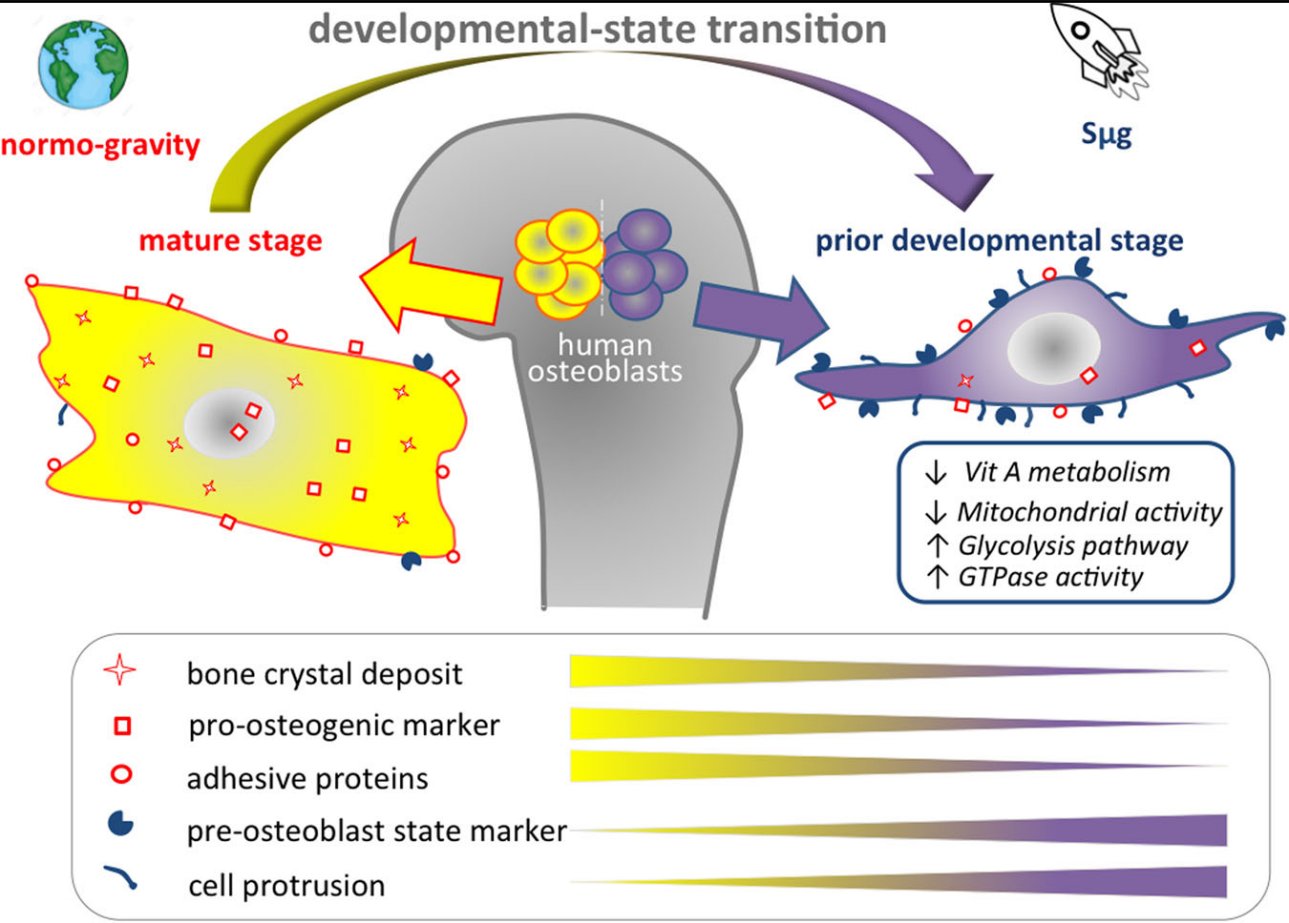
Graphical sketch of the effect of Sμg treatment on hpOBs. Microgravity uncovers a developmental state transition on human osteoblasts. Upon simulated microgravity exposition human primary osteoblasts assume spindle-shape morphology reverting the mature phenotype and their specialized functions. A mature osteoblast cell loses levels of bone crystal deposit, osteogenic markers, and adhesive proteins, enhancing prior developmental state features (glycolysis, pre-osteoblast state marker, and cell protrusions), which in turn allows it to acquire motility properties.

Overall, this investigation gives a functional and morphological overview of how hpOBs receive an impulse to dedifferentiate by gravitational unloading. Present findings suggest that Sμg could be employed to promote cell dedifferentiation and potentially be addressed to trans-differentiate to alternative cell types (Fig. 6). Clearly, the complexity of dedifferentiation processes deserves further studies to understand more accurately how they work and eventually to harness them for use in regenerative medicine. On the other hand, ex vivo, screening of unloading treatments on hpOBs from osteodegenerative patients could be of help for developing a rational new therapeutically biomechanical strategies for the treatment of skeletal disorders on Earth and to assure safe and effective aerospace missions.

## Materials and methods

### Simulation of microgravity by RPM

The desktop RPM system (Dutchspace, The Netherlands) was used for carrying out the investigation on the influence of the force of gravity on eukaryotic cells^50^. All experiments were carefully planned according to procedures previously described^51^. Briefly, the rotating frame of the desktop RPM was placed inside an ordinary cell culture CO_2_ incubator. The software responsible for controlling the motion of RPM employed a tailored algorithm, which rotated with a random speed in such a way that the mean gravity vector reliably converged to zero over time, and it concurrently reduced fluid motion in the culture flask. In order to avoid artifacts and to minimize centrifugal acceleration, the samples were compactly placed around the center of rotation. Cell samples were carefully processed for in vitro cultivation: the culturing media were accurately sealed with a transpiring membrane, which was pressed to completely remove air bubbles from the culture chamber. Control samples were cultured and processed in the same manner. Plates were placed beside the RPM machine so that all samples shared identical culture conditions. Since most of the ground-based microgravity research platforms are not vibration free, high-performance microscopy has not been applicable in Live Cell Imaging under RPM. Therefore, studies involving cell imaging have been carried out after chemical fixation of the cell. This approach implies a series of static shots, which reproduce dynamic events occurring in cells in response to Sμg exposure.

### Patient characteristics

Patients selected for the study were screened to exclude any association with clinical or pathological variables (carefully grouped on the bases of BMD parameters obtained by dual-energy X-ray absorption T-score greater that −1). No patient showed any sign of bone or joint disease or autoimmune disorder. Informed consent was obtained from all patients. The biopsies were collected from high energy fractures of femoral head of healthy patients during hip replacement surgery. Biopsy samples were taken from selected patients to set up human primary cells cultures in vitro. All procedures were approved by the Institutional Review Board of Policlinico Tor Vergata Hospital, Rome, Italy (approval reference number #85/12). Since no effects of sex on adaptation to space had been previously observed^52^, we decided to analyze osteoblasts from both male and female patients in order to determine average effects.

Experiments were performed in accordance with relevant guidelines and regulations. hpOBs were isolated from 3 donors (2 males and 1 female, average age 53 years, namely, female 52 years, males 49 and 59 years) undergoing hip replacement surgery and used to perform separate experiments investigating the effects of Sμg vs. normo-gravity conditions.

### Isolation and culture of primary human OB cells

Primary cultures of osteoblasts were isolated from the cancellous bone of healthy patients with high-energy femoral fracture. The bone tissue was minced, thoroughly washed to remove any remaining soft tissue, and placed in 6-well plates to initiate explant cultures. The culture medium consisted of DMEM/F12 (Biowest, Nuaillé, FR) supplemented with 15% FBS, 50 μg/ml gentamicin, and 0.08% Fungizone, penicillin streptomycin (Sigma Chemical Co., St. Louis, MO, USA), and amphotericin B (biowest) and was changed twice per week. Cells were treated to select and isolate homogeneous populations of osteoblasts according to previously reported methods^53^. For further details, see Supplementary Information.

### Validation of hpOBs

To assess the quality of each cell purification a fraction of the purified cells were inspected. Using immunochemistry analysis and morphological inspection the isolated primary cells were observed to be homogeneous and appropriate for osteoblasts, expressing BMP-2 RUNX2 and RANK L. For further details, see Supplementary Information. Cells were seeded in separate flasks, so that once cells reached the confluence, these could feasibly follow three different experimental steps. One subset of samples were devoted to cells inspection for the quality control (for providing cell baseline before treatment), a second subset was dedicated to the simulated microgravity treatment and the last one was employed for the nomogravity control conditions. Only the subset of samples that succeed this quality check they were employed to test the impact of gravity on hpOB differentiation.

### Immunostaining

Cell samples were washed with PBS and chemically fixed with 4% paraformaldehyde (PF, 4% in PBS). For immuno-cytostaining analysis, cells were treated, first for 30 min with EDTA citrate/tween 20, pH 7.8 at 95 °C, and then incubated with mouse monoclonal anti-RUNX2 antibody for 60 min (1 µg/ml, clone EPR14334, AbCam), rabbit monoclonal anti-RANKL for 60 min (1 µg/ml, clone 12A668, AbCam), mouse monoclonal anti-BMP-2 antibody for 60 min (1 µg/ml, clone 65529.111, AbCam), rabbit monoclonal anti-CD44 for 60 min (1 µg/ml, clone EPR1013Y, AbCam). Washings were performed with PBS/Tween20 pH 7.6 (UCS Diagnostic, Rome, Italy); reactions were revealed by using horseradish peroxidase—3,3′ diaminobenzidine (DAB) Detection Kit (UCS Diagnostic, Rome, Italy). To assess the background of immuno-staining, we included a negative control for each reaction by incubating the sections with secondary antibodies (HRP) and a detection system (DAB). The immuno-histochemical reaction was semi-quantitatively evaluated by assigning a score from 1 to 3 according to the number of positive cells. Results were showed as percentage of positive cells.

### Transmission electron microscopy

Cell samples were chemically fixed and processed for TEM to inspect the deposits of HA crystals. In detail, cells were fixed in 4% (v/v) *p*-formaldehyde and post-fixed in 2% osmium tetroxide. After washing with 0.1 M phosphate buffer, the sample was dehydrated by a series of incubations in 30, 50, and 70% (v/v) ethanol. Dehydration was continued by incubation steps in 95% ethanol, absolute ethanol, and propylene oxide, after which samples were embedded in Epon (Agar Scientific, Stansted, Essex CM24 8GF, UK). Eighty micrometer ultra-thin sections were mounted on copper grids and examined with a transmission electron microscope (Model 7100FA, Hitachi, Schaumburg, IL, USA). For EDX microanalysis, ultra-thin sections were mounted on copper grids. EDX spectra of HA crystal within osteoblasts were acquired with an EDX detector (Thermo Scientific, Waltham, MA, USA) at an acceleration voltage of 75 KeV and magnification of 12.000 Spectra were semi quantitatively analyzed by the Noram System Six software (Thermo Scientific, Waltham, MA, USA) using the standardless Cliff–Lorimer k-factor method.

Percentage of both osteoblasts and mesenchymal-like cells was evaluated by counting the number of them over a total of 100 cells.

### Assays to evaluate the effect of simulated microgravity on cell number

To determine the impact of Sμg on cell number we evaluate (i) the number of cells with intact membrane, (ii) the number of mitochondrial-active cells, and (iii) protein cell content as previously described by us^39^. For further details, see Supplementary section.

### Scratch wound healing assay

The in vitro model of wound healing was employed for examining the ability of hpOBs to migrate under Sμg exposition (migrations of SAOS II cell was also examined, see Supplementary Information). Cells were seeded on gelatin-coated glass slides and growth till confluence in regular medium. Cell monolayers were wounded by scratching monolayer with a sterile p200 pipette tip. Afterward, wounded monolayers were thoroughly washed with PBS, and the serum-free culture media were returned to the cells. Cell migration occurred in serum-free media guaranteed a growth arrest and to get read of proteases and other soluble factor which would interfere with cell mobility assay. Capture of the images during the cell migration close to the wound were taken employing Nikon reverse microscopy equipped with stacked IMX214 BSI sensor by Sony, upgraded type 1/3.06 imaging module and a wide f/2.0 aperture Shoot 50-megapixel photos with the Ultra-HD “multi-shot feature”. To derive cell migration rate widths of the wound gaps were measured by an electronic micrometer scale, and the results were plotted on a graph (according the established method previously described)^54^. Migration of the cells into wounded areas of the monolayer by measuring the distance traveled toward the center of the wound area (percentage of overgrown area) over the time. To measure the distance between the leading edges of wounded region lacking cells a free software was employed, freely retrieved from website https://imagej.nih.gov/ij/.

### MS sample preparation

At day 5 Sμg-treated and control cells in three biological replicates were washed in phosphate saline buffer. Cellular suspensions were centrifuged at 1500 × *g* for 5 min. The supernatant was discarded and cell pellet was resuspended in lysis buffer (i.e., 7 M urea, 2 M thiourea, 4% w/v CHAPS, 40 mM Tris-HCl, 0.1 mM EDTA, 1 mM DTT, 50 mM NaF, 0.25 mM Na_2_VO_4_).

For an accurate determination of total protein concentration, 2D-Quant Kit (E Healthacare) was used. Aliquots of 150 µg for each sample were loaded per lane and separated through a 16–8% linear gradient polyacrylamide gel. Each lane was cut into 72 slices about 2 mm thick and these were subjected to in-gel trypsin digestion^55^.

### LC-MS/MS analysis

Peptide extracts were analyzed using a split-free nano-flow liquid chromatography system (EASY-nLC II, Proxeon, Odense, Denmark) coupled with a 3D-ion trap (model AmaZon ETD, Bruker Daltonik, Germany) equipped with an online ESI nanosprayer (the spray capillary was a fused silica capillary, 0.090 mm OD, 0.020 mm ID) in positive ion mode. For all experiments, a 15 μL sample volume was loaded by the autosampler onto a homemade 2 cm fused silica pre-column (100 μm I.D.; 375 μm O.D.; Reprosil C18-AQ, 5 μm, Dr. Maisch GmbH, Ammerbuch-Entringen, Germany). Sequential elution of peptides was accomplished using a flow rate of 300 nL/min and a linear gradient from Solution A (2% acetonitrile; 0.1% formic acid) to 50% of Solution B (98% acetonitrile; 0.1% formic acid) in 40 min over the pre-column online with a homemade 15 cm resolving column (75 μm I.D.; 375 μm O.D.; Reprosil C18-AQ, 3 μm, Dr. Maisch GmbH, Ammerbuch-Entringen, Germany). The acquisition parameters for the mass spectrometer were as follows: dry gas temperature, 220 °C; dry gas, 4.0 L/min; nebulizer gas, 10 psi; electrospray voltage, 4000 V; high-voltage end-plate offset, −200 V; capillary exit, 140 V; trap drive: 63.2; funnel 1 in 100 V out of 35 V and funnel 2 in 12 V out of 10 V; ICC target, 200,000, and maximum accumulation time, 50 ms. The sample was measured with the Enhanced Resolution Mode at 8100 *m*/*z* per second (which allows mono-isotopic resolution up to four charge stages), scan range from *m*/*z* 300 to 1500, 5 spectra averaged, and rolling average of 1. The “Smart Decomposition” was set to “auto”.

Label-free quantitative analyses were performed in biological triplicates by using the spectral counting method based on normalized emPAI as described by others^56^. In detail, for each protein the percentage was calculated as follows:

Protein content (%) = emPAI/ΣemPAI × 100

Statistically significant differences were identified by unpaired *t*-Student's test.

### MS data analysis

Compass DataAnalysis 4.0 software (Bruker Daltonics) was used for data processing. Generated mgf files were then merged per lane and used for database search (SwissProt, version 20150612) through the MASCOT Daemon application included in an in-house MASCOT server (version 2.5, Matrix Science, London, UK) with the following constraints: taxonomy = Homo sapiens (20,207 sequences); enzyme = trypsin; missed cleavage = 1; peptide and fragment mass tolerance = ± 0.3 Da; fixed modifications = Carbamidomethyl (Cys); variable modifications = Oxidation(Met).

Label-free quantitative analyses were performed in three biological triplicates by using the spectral counting method based on normalized emPAI as previously described^56^.

To obtain a comprehensive description of the overrepresented biological processes and functional-related groups of proteins within our data set, a Bioinformatic Gene Ontology analysis was performed by the online FunRich software 3.0 (www.funrich.org). As background, the default Homo sapiens genome was used.

### Metabolomic extraction

1 **×** 10^6^ cells from each treatment (three biological replicates × three technical replicates × two conditions; *n* = 18) were first subjected to three freeze-melt cycles (freezing in ice for 5 min, melting at 37 °C for 5 min; for five times). Next, 400 μl of freezing methanol and 600 μl of freezing chloroform were added to the cells. Samples were vortexed for 30 min at max speed at 4 °C. The next day, samples were centrifuged at 16,000 × *g* for 15 min at 4 °C, supernatants were evaporated to dryness using an SPD2010–230 SpeedVac Concentrator (Thermo Savant, Holbrook, USA). When samples were completely dried, 60 μl of 5% formic acid was added to the dried residue and vigorously vortex mixed.

### UHPLC-HRMS

Twenty microliters of samples were injected into an ultra-high-performance liquid chromatography (UHPLC) system (Ultimate 3000, Thermo) and run on a positive mode: Samples were loaded onto a Reprosil C18 column (2.0 mm × 150 mm, 2.5 μm—Dr. Maisch, Germany) for metabolite separation. Chromatographic separations were achieved at a column temperature of 30 °C and flow rate of 0.2 ml/min. For positive ion mode (+) MS analyses, a 0–100% linear gradient of solvent A (ddH2O, 0.1% formic acid) to B (acetonitrile, 0.1% formic acid) was employed over 20 min, returning to 100% A in 2 min and a 6-min post-time solvent A hold. Acetonitrile, formic acid, and HPLC-grade water and standards (≥98% chemical purity) were purchased from Sigma-Aldrich. The UHPLC system was coupled online with a mass spectrometer Q Exactive (Thermo) scanning in full MS mode (2 μscans) at 70,000 resolution in the 67–1000 *m*/*z* range, target of 1106 ions and a maximum ion injection time (IT) of 35 ms 3.8 kV spray voltage, 40 sheath gas, and 25 auxiliary gas, operated in positive ion mode. Source ionization parameters were: spray voltage, 3.8 kV; capillary temperature, 300 °C; and S-Lens level, 45. Calibration was performed before each analysis against positive or negative ion mode calibration mixes (Piercenet, Thermo Fisher, Rockford, IL) to ensure sub ppm error of the intact mass. Metabolite assignments were performed using computer software (Maven,18 Princeton, NJ), upon conversion of raw files into mzXML format through MassMatrix (Cleveland, OH).

### Data elaboration and statistical analysis

Replicates were exported as mzXML files and processed through MAVEN.52; MS chromatograms were elaborated for peak alignment, matching and comparison of parent and fragment ions, and tentative metabolite identification (within a 2 ppm mass deviation range between observed and expected results against the imported KEGG database).

Data are presented as mean ± SD. The difference between the two groups was compared with unpaired *t*-test, *p < 0.05 was considered significant. A one-way analysis of variance (ANOVA) was performed and followed by Tukey’s honestly significant difference test. Asterisk represents data significantly different from the respective control. Statistics were calculated by GraphPad Prism software, version 5.0 (La Jolla, CA).

## Electronic supplementary material

SUPPLEMENTARY FIGURE 1

SUPPLEMENTARY TABLE 1

Supplementary Information
