# Supplementary figures and images for: Simulated microgravity induces a cellular regression of the mature phenotype in human primary osteoblasts
Source: Cell Death Discov. 2018 May 10;4:59. doi: 10.1038/s41420-018-0055-4 (PMC5945613; doi:10.1038/s41420-018-0055-4)

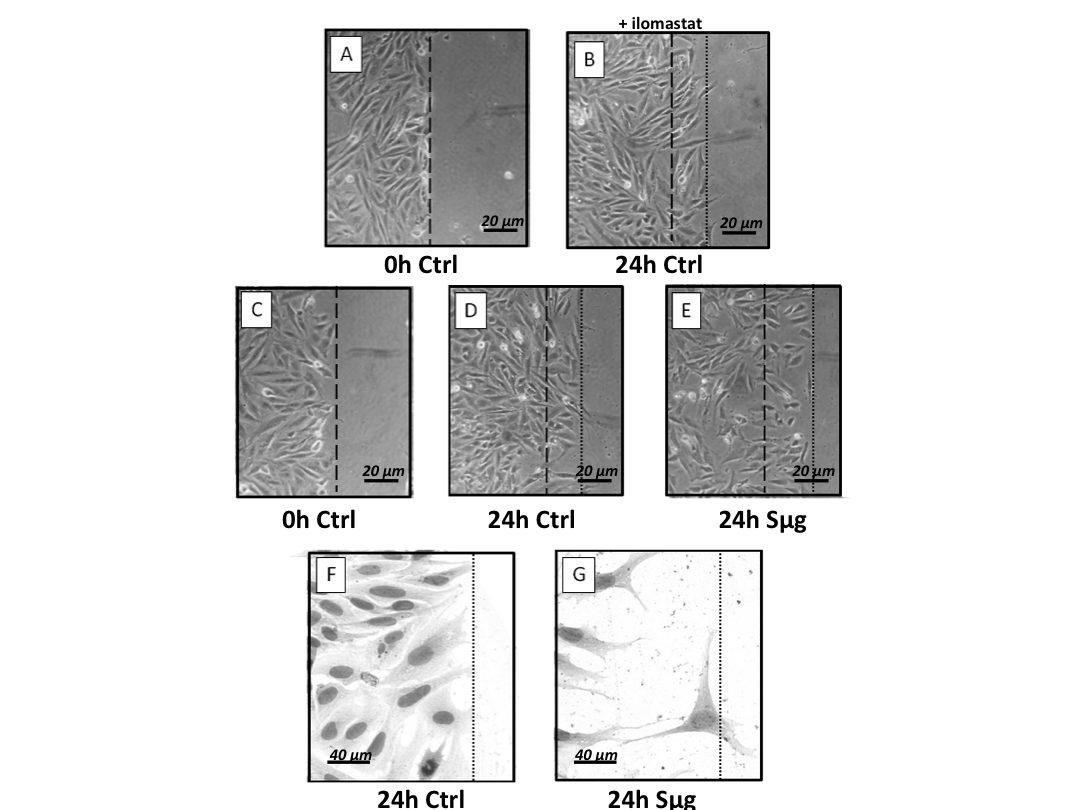

Supplement: Supplementary file 1 — SUPPLEMENTARY FIGURE 1 [file 41420_2018_55_MOESM1_ESM.tif]
