# Supplementary material for: Simulated microgravity induces a cellular regression of the mature phenotype in human primary osteoblasts
Source: Cell Death Discov. 2018 May 10;4:59. doi: 10.1038/s41420-018-0055-4 (PMC5945613; doi:10.1038/s41420-018-0055-4)
Supplement: Supplementary file 2 — SUPPLEMENTARY TABLE 1 [file 41420_2018_55_MOESM2_ESM.docx]

| **Molecular function enrichment item (FunRich)** | **Protein subclass** | **Protein Name** | **Biological Function** | **Trend** |
| --- | --- | --- | --- | --- |
| **osteoblast differentiation** | mineralization  proteins | Alkaline phosphatase, tissue-nonspecific isozyme | This isozyme may play a role in skeletal mineralization. | ↓ |
|  |  | Alpha-crystallin B chain | Has chaperone-like activity, preventing aggregation of various proteins under a wide range of stress conditions.  it is a novel osteogenic marker (Graneli et al 2014) | ↓ |
|  | Tropomyosin alpha-4 chain | | Binds to actin filaments in muscle and non-muscle cells. Plays a central role, in association with the troponin complex, in the calcium dependent regulation of vertebrate striated muscle contraction. Smooth muscle contraction is regulated by interaction with caldesmon. In non-muscle cells is implicated in stabilizing cytoskeleton actin filaments. Binds calcium | ↓ |
|  | **migration proteins** | Clathrin heavy chain 1 | Clathrin is the major protein of the polyhedral coat of coated pits and vesicles. Acts as component of the TACC3/ch-TOG/clathrin complex proposed to contribute to stabilization of kinetochore fibers of the mitotic spindle by acting as inter-microtubule bridge. The TACC3/ch-TOG/clathrin complex is required for the maintenance of kinetochore fiber tension. Plays a role in early autophagosome formation | ↓ |
|  |  | Ras-related protein R-Ras2 | It is a plasma membrane-associated GTP-binding protein with GTPase activity. It’s important for osteoblast differentiation and cell migration | ↓ |
|  |  | Fibroblast growth factor receptor 2 | Tyrosine-protein kinase that acts as cell-surface receptor for fibroblast growth factors and plays an essential role in the regulation of cell proliferation, differentiation, migration and apoptosis. Plays an essential role in the regulation of osteoblast differentiation, proliferation and apoptosis, and is required for normal skeleton development. | ↓ |
| **(i)Adhesion proteins** | **Integrin binding proteins** | Talin | Probably involved in connections of major cytoskeletal structures to the plasma membrane. High molecular weight cytoskeletal protein concentrated at regions of cell-substratum contact and, in lymphocytes, at cell-cell contacts | ↓ |
|  |  | Zyxin | Adhesion plaque protein. Binds alpha-actinin and the CRP protein. Important for targeting TES and ENA/VASP family members to focal adhesions and for the formation of actin-rich structures. May be a component of a signal transduction pathway that mediates adhesion-stimulated changes in gene expression | ↓ |
|  | **integrins** | integrin beta 1 | Involved in osteoblast compaction through the fibronectin fibrillogenesis cell-mediated matrix assembly process and the formation of mineralized bone nodules | ↓ |
|  |  | Integrin alpha-3 | It’s a receptor for fibronectin, laminin, collagen, epiligrin, thrombospondin and CSPG4. Integrin alpha-3/beta-1 provides a docking site for FAP (seprase) at invadopodia plasma membranes in a collagen-dependent manner and hence may participate in the adhesion, formation of invadopodia and matrix degradation processes, promoting cell invasion | ↓ |
| **(ii)Protrusion dynamic actin network** | **Arp2/3 complex**  **proteins** | Actin-related protein 2/3 complex subunit 1B (p41) | subunits involved in regulation of actin polymerization and together with an activating nucleation-promoting factor (NPF) mediates the formation of branched actin networks | UN |
|  |  | Actin-related protein 2/3 complex subunit 2 (ARPC2/p34) |  | ↓ |
|  |  | Actin-related protein 2/3 complex subunit 3 (ARPC3/p21) |  | UM |
|  |  | Actin-related protein 2/3 complex subunit 4 (p20) |  | ↑ |
|  |  | Actin-related protein 2/3 complex subunit 5 (p16) |  | ↑ |
|  |  | Actin-related protein 2  (ARP2) | Functions as ATP-binding component of the Arp2/3 complex which is involved in regulation of actin polymerization and together with an activating nucleation-promoting factor (NPF) mediates the formation of branched actin networks. Seems to contact the pointed end of the daughter actin filament. | UN |
|  |  | Actin-related protein 3 (ARP3) |  | ↓ |
|  | **cytoskeletal organization proteins** | ENAH  Protein enabled homolog | Ena/VASP proteins are actin-associated proteins involved in a range of processes dependent on cytoskeleton remodeling and cell polarity such as axon guidance and lamellipodial and filopodial dynamics in migrating cells. ENAH induces the formation of F-actin rich outgrowths in fibroblasts. Acts synergistically with BAIAP2-alpha and downstream of NTN1 to promote filipodia formation | ↓ |
|  |  | VASP  Vasodilator-stimulated phosphoprotein | VASP promotes actin filament elongation. It protects the barbed end of growing actin filaments against capping and increases the rate of actin polymerization in the presence of capping protein. VASP stimulates actin filament elongation by promoting the transfer of profilin-bound actin monomers onto the barbed end of growing actin filaments. Plays a role in actin-based mobility of Listeria monocytogenes in host cells. | UM |
|  |  | cofilin-1 | Binds to F-actin and exhibits pH-sensitive F-actin depolymerizing activity. Regulates actin cytoskeleton dynamics. Important for normal progress through mitosis and normal cytokinesis. Plays a role in the regulation of cell morphology and cytoskeletal organization. | ↓ |
|  |  | profilin | Binds to actin and affects the structure of the cytoskeleton. At high concentrations, profilin prevents the polymerization of actin, whereas it enhances it at low concentrations. By binding to PIP2, it inhibits the formation of IP3 and DG. Inhibits androgen receptor (AR) and HTT aggregation and binding of G-actin is essential for its inhibition of AR ([Shao J](https://www.ncbi.nlm.nih.gov/pubmed/?term=Shao%20J%5BAuthor%5D&cauthor=true&cauthor_uid=18573880). *et al*. Phosphorylation of profilin by ROCK1 regulates polyglutamine aggregation. [*Mol Cell Biol*](https://www.ncbi.nlm.nih.gov/pubmed/18573880)*.* 28(17):5196-208 (2008).) | ↓ |
|  |  | fascin | Organizes filamentous actin into bundles. Plays a role in the organization of actin filament bundles and the formation of microspikes, membrane ruffles, and stress fibers. Important for the formation of a diverse set of cell protrusions, such as filopodia, and for cell motility and migration. | UM |
|  |  | radixin | Probably plays a crucial role in the binding of the barbed end of actin filaments to the plasma membrane. | UM |
|  |  | Myosin-10 | Cellular myosin that appears to play a role in cytokinesis, cell shape, and specialized functions such as secretion and capping. During cell spreading, plays an important role in cytoskeleton reorganization, focal contacts formation (in the central part but not the margins of spreading cells), and lamellipodial extension; this function is mechanically antagonized by MYH9. | UN |
| **(iii)RhoGTP signaling** | **Rho GTPases** | RHOA  transforming protein rhoA precursor | Regulates a signal transduction pathway linking plasma membrane receptors to the assembly of focal adhesions and actin stress fibers. Involved in a microtubule-dependent signal that is required for the myosin contractile ring formation during cell cycle cytokinesis. Plays an essential role in cleavage furrow formation. Required for the apical junction formation of keratinocyte cell-cell adhesion. | UM |
|  |  | RHO G  Rho related GTP-binding protein RhoG precursor | Required for the formation of membrane ruffles during macropinocytosis. Plays a role in cell migration and is required for the formation of cup-like structures during trans-endothelial migration of leukocytes. | UM |
|  |  | RAC1  Ras-related C3 botulinum toxin substrate 1 | Plasma membrane-associated small GTPase which cycles between active GTP-bound and inactive GDP-bound states. In its active state, binds to a variety of effector proteins to regulate cellular responses such as secretory processes, phagocytosis of apoptotic cells, epithelial cell polarization and growth-factor induced formation of membrane ruffles | UM |
|  | **negative regulators of GTPases** | ARHGDIA  Rho GDP-dissociation inhibitor 1 | Controls Rho proteins homeostasis. Regulates the GDP/GTP exchange reaction of the Rho proteins by inhibiting the dissociation of GDP from them, and the subsequent binding of GTP to them. | ↓ |
|  |  | ARHGDIB  Rho GDP-dissociation inhibitor 2 | Regulates the GDP/GTP exchange reaction of the Rho proteins by inhibiting the dissociation of GDP from them, and the subsequent binding of GTP to them (PubMed:8356058, PubMed:7512369). Regulates reorganization of the actin cytoskeleton mediated by Rho family members. | UN |
|  |  | ARHGEF18  Rho guanine nucleotide exchange factor 18 | Acts as guanine nucleotide exchange factor (GEF) for RhoA GTPases. Its activation induces formation of actin stress fibers. | UN |
|  | **activator of GTPase** | RHG01  Rho GTPase-activating protein 1 | GTPase activator for the Rho, Rac and Cdc42 proteins, converting them to the putatively inactive GDP-bound state. Cdc42 seems to be the preferred substrate. | UM |
|  |  | ROCK2  Rho associate protein kinase 2 | Protein kinase which is a key regulator of actin cytoskeleton and cell polarity. Involved in regulation of smooth muscle contraction, actin cytoskeleton organization, stress fiber and focal adhesion formation, neurite retraction, cell adhesion and motility | UN |
| **vitamin A metabolism** | **negative regulators of vit A metabolisms** | calreticulin | Calcium-binding chaperone that promotes folding, oligomeric assembly and quality control in the endoplasmic reticulum (ER) via the calreticulin/calnexin cycle. It can negatively regulate RAR function | ↑ |
|  |  | HMGB1  High mobility group protein B1 | Multifunctional redox sensitive protein with various roles in different cellular compartments. In the nucleus is one of the major chromatin-associated non-histone proteins and acts as a DNA chaperone involved in replication, transcription, chromatin remodeling, V(D)J recombination, DNA repair and genome stability. RA inhibits the release of HMB1 in TNFα activated endothelilal cells | ↑ |
|  | **positive regulators of vit A metabolisms** | HMG A1  High mobility group protein HMG-I/HMG-Y | Interact selectively and non-covalently with retinoic acid receptor | ↓ |
|  |  | ACTN4  Alpha-actinin-4 | F-actin cross-linking protein which is thought to anchor actin to a variety of intracellular structures. ACTN4 modulates transcriptional activity of RA receptor (Khurana et al., 2012. Familial Focal Segmental Glomerulosclerosis (FSGS)-linked α-Actinin 4 (ACTN4) Protein Mutants Lose Ability to Activate Transcription by Nuclear Hormone Receptors) | ↓ |
|  |  | Guanine nucleotide-binding protein GI/GS/GT subunit beta-1 | Guanine nucleotide-binding proteins (G proteins) are involved as a modulator or transducer in various transmembrane signaling systems. The beta and gamma chains are required for the GTPase activity, for replacement of GDP by GTP, and for G protein-effector interaction. Rhodopsin mediated signaling pathway | ↓ |
