# Supplementary material for: Simulated microgravity induces a cellular regression of the mature phenotype in human primary osteoblasts
Source: Cell Death Discov. 2018 May 10;4:59. doi: 10.1038/s41420-018-0055-4 (PMC5945613; doi:10.1038/s41420-018-0055-4)
Supplement: Supplementary file 3 — Supplementary Information [file 41420_2018_55_MOESM3_ESM.docx]

**Supplementary Information**

**Supplementary Data**

***Effect of Sμg on SOASII oteoblasts: under simulated microgravity cell migration of osteosarcoma cells is MMP independent***

As referenced osteoblast cell line Sarcoma osteogenic Saos-2 (ATCC^®^ HTB-85^™^) have been employed. As also reported by others,^(11)^ on Earth osteosarcoma cell migration was dependent on matrix metalloproteinase (MMP) activity (data not shown). By contrast, Fig. 1S indicates that under Sμg conditions the malignant osteoblast migration to the site of injury occurred even in the presence of 26 μM ilomastat (an irreversible broad-range inhibitor for MMPs). It indicates that the migration properties of osteosarcoma cells depend on the gravitational force, involving distinct proteolytic sets of enzymes.

Furthermore, a 24 hours-treatment of Sμg turned out to be an osteotropic agent which enhances SAOS-II cell-motility, as demonstrated by the difference of cell-migration rates between normo- (3.2±0.1 μm/h) and micro-gravity conditions (3.7±0.05 μm/h). Therefore, on the basis of the wound healing assay on SAOS-II cells we observe that Sμg *a*) increases the migration velocity by 15%, *b*) renders the cell migration process independent on MMPs proteolytic activities (Fig. 1S), c) induces a loosening of the confluence layer, motility becoming individual rather than collective (Figs. 1S (F-G). Since proteolytic and non-proteolytic cell motility involve distinct migration strategies,^(12,13)^ these findings suggest that under Sμg conditions migration of osteosarcoma cell line occurs through a distinct migration strategy, which is sensitive to the gravitational force. Of note, Fig. 1S clearly shows that under Sμg cell layers lose confluence (likely due to a decrease of cell-cell contacts) and the cell migration becomes individual (Fig. 1S (G)).

**Supplementary Materials and methods**

**Isolation and culture of primary human osteoblasts**

Briefly, after dissection, trabecular bone chips were repeatedly washed with PBS at 37°C for 2 h in shaking conditions. Then, two distinct enzymatic digestions were repeated and performed at 37°C. The first digestion employed 1mg/ml Trypsin from porcine pancreas ≥ 60 U/mg (SERVA Electrophoresis GmbH Heidelberg, DE) resuspended in PBS buffered at pH 7.2. After washing, trypsinized bone chips underwent to repeated digestions with a second type of protease employing 2.5 mg/ml Collagenase NB 4G Proved grade ≥ 0.18U/mg (SERVA Electrophoresis GmbH, Heidelberg, DE) in PBS buffer with Calcium and Magnesium. The supernatants from the 4th bone-chips digestion was collected and centrifuged at 310 RCF for 5’. The cell pellets was resuspended in DMEM with 15% FBS, thus cell were then grown in low calcium media, supplemented with fetal bovine serum (10%; Intergen, Purchase, NY, USA), penicillin (50 U/ml), and streptomycin *(50* pg/ml). When the cultures reached confluence in 3-5 weeks, the bone chips were removed and the cellular outgrowths treated with trypsin (0.05%) and EDTA (0.02%) to prepare single cell suspensions. All cells were incubated at 37°C and 5% CO_2_. Upon confluence, cells were detached from the plates by trypsinization, counted and subcultured at a density of 5 000 cells/cm^2^ for three passages. Osteoblast proliferation was compared between different tissue sources at passage one. Third passage cells were used in all other experiments.

***Quality check for human primary osteoblasts***

To provide a baseline before the treatment and to assess the quality of each cell purification a fraction of the purified cells were inspected. Using immunochemistry analysis and morphological inspection the isolated primary cells were observed to be homogeneous and appropriate for osteoblasts, expressing BMP-2 RUNX2 and RANK L. Briefly, cells were seeded in a monolayer at 40 000 cells/cm^2^ and cultured until the confluence was reached. Primary osteoblasts were cultured for two weeks in osteogenic medium (OGM) containing 10 mM biglycerol phosphate, 50 µM ascorbic acid, 25 ng/ml bone morphogenetic protein-2 (BMP-2; R&D Systems) in 10% serum containing alpha MEM. After two weeks of incubation, the cells were assessed for alkaline phosphatase activity as an indicator of differentiation. Morphological inspection was carried out to assess HA precipitated crystals. Osteoblast phenotype was characterised by immunohistochemistry as BMP-2, RUNX-2 and RANK-L. Osteoblast cell lysates were generated using standard lysis buffers. The total protein content in the cells was measured by BCA and Biuret assay. Whereas alkaline phosphatase activity was measured in normalized samples using p-nitrophenyl phosphate (p-NPP; Pierce, Rockford, IL, USA) in a 1 M diethanolamine buffer at pH 9.8. Absorbance in control and treated cells was measured at 405 nm.

***Assays to evaluate the effect of simulated microgravity on cell number***

The number of viable cells were assessed using the trypan blue dye exclusion procedure. When required, cells were detached by incubating plates with trypsin/versine solution for 10 minutes at 37°C. Cell solution was mixed 1:1 with trypan blue stain (Invitrogen) and cells with intact membranes (viable cells) were counted using a haemocytometer device under an optical microscope (Nikon Eclipse TE 2000-5). To determine the number of viable metabolically active cells (i.e. possessing active mitochondria), the cell titer 96 AQ cell colorimetric assay (Promega) was employed, following the manufacturers’ instructions. After incubation for four hours, MTS formazan soluble product was measured at absorbance 490 nm with a Tecan Spark 10N spectrophotometer reader. Spectrophotometric determination of cell protein content was evaluated by the biuret colorimetric analysis of peptides and the bicinchoninic acid (BCA) method, with readings at wavelengths of 310 nm and 562 nm, respectively (Biuret reagent kit, Sigma-Aldrich; BCA Protein Assay Kit, The Thermo Scientific Pierce).

For all three assays, standard curve validated that the absorbance (at 490 nm, 310 nm or 562 nm, respectively) read were directly proportional to the number of living cells in culture. With all three methods, the effect of the treatment was reported in terms of percentages, as follows: the percentage calculation was based on the ratio of the mean value of Sμg-treated samples to the mean value of normo-gravity (control) samples. The statistical significance of data was determined using the Student’s t-test.( as reported by us^39^)

**Supplementary legends**

**Fig. 1S** SAOS II cell migration under microgravity becomes. **I)** MMP independent**.** (A) Phase contrast image of cell monolayer recorded immediately after the scratch (t0).(B) The wound after 24h microgravity exposition in the presence of 26 μM ilomastat(MMP irreversible inhibitor). In microgravity environment cell motility occurs even in the presence of 26 μM ilomastat. **II)** faster (C) scratched cell monolayer at t0. (D) 24h post the wound under normo-gravity. (E) 24h post the wound under microgravity (microscopic magnification 4X). **III)** not-through sheet of cells (F) and (G): zoom on leading edges on BMP2 Immunostained cells 24h normo- and micro-gravity, respectively (magnification 40X).

**Table 1S** . Biological functions of the proteome which is differential regulated upon Sμg treatment. The table shows trends of osteoblast-differentiation proteins differentially regulated in abundance. ↓, indicates microgravity induced down regulation of proteins. ↑, indicates microgravity-induced up-regulation of protein. (UN), indicates Unique proteins in normo-gravity and (UM)refers to Unique proteins found Sμg proteome. For each protein its biological functions is specified.
